# Brain Correlates of Experience-Dependent Changes in Stimulus Discrimination Based on the Amount and Schedule of Exposure

**DOI:** 10.1371/journal.pone.0101011

**Published:** 2014-06-26

**Authors:** Matthew E. Mundy, Paul E. Downing, Robert C. Honey, Krish D. Singh, Kim S. Graham, Dominic M. Dwyer

**Affiliations:** 1 Wales Institute of Cognitive Neuroscience (WICN), School of Psychology, Cardiff University, Cardiff, United Kingdom; 2 Wales Institute of Cognitive Neuroscience (WICN), School of Psychology, Bangor University, Gwynedd, United Kingdom; 3 School of Psychology, Cardiff University, Cardiff, United Kingdom; 4 Cardiff University Brain Imaging Research Centre (CUBRIC), Cardiff University, Cardiff, United Kingdom; 5 Monash University, School of Psychological Sciences, Melbourne, Victoria, Australia; 6 School of Psychology, University of New South Wales, Sydney, New South Wales, Australia; University of Leuven, Belgium

## Abstract

One product of simple exposure to similar visual stimuli is that they become easier to distinguish. The early visual cortex and other brain areas (such as the prefrontal cortex) have been implicated in such perceptual learning effects, but the anatomical specificity within visual cortex and the relationship between sensory cortex and other brain areas has yet to be examined. Moreover, while variations in the schedule (rather than merely the amount) of exposure influence experience-dependent improvement in discrimination, the neural sequelae of exposure schedule have not been fully investigated. In an event-related fMRI study, participants were exposed to confusable pairs of faces, scenes and dot patterns, using either intermixed or blocked presentation schedules. Participants then performed same/different judgements with exposed and novel pairs of stimuli. Stimulus independent activation, which was correlated with experience-dependent improvement in discrimination, was seen in frontal areas (e.g. frontal and supplementary eye fields and dorsolateral prefrontal cortex) and in early visual cortex (V1-4). In all regions, the difference in activation between exposed and novel stimuli decreased as a function of the degree of discrimination improvement. Overall levels of BOLD activation differed across regions, consistent with the possibility that, as a consequence of experience, processing shifts from initial engagement of early visual regions to higher order visual areas. Similar relationships were observed when contrasting intermixed with blocked exposure, suggesting that the schedule of exposure primarily influences the degree of, rather than the mechanisms for, discrimination performance.

## Introduction

It is well established that simply giving participants exposure to stimuli results in an improvement in the subsequent ability to discriminate between those stimuli (for reviews see [1]–[3]). This experience-dependent change in discriminability is one example of perceptual learning which Gibson defined as “any relatively permanent and consistent change in the perception of a stimulus array, following practice or experience with this array” (Gibson, [4], p. 29; see also Goldstone, [2], p. 585). Following Gibson, the study of experience-dependent changes in discriminability has been pursued in two partially independent traditions. One, influenced by an associative perspective, has focused on the role of stimulus comparison using analogous procedures in animals and humans, utilising a range of relatively complex stimuli (e.g., flavours [5], [6]; faces [7]; checkerboards [8], [9]; visual scenes [10]). The other, being generally characterized by the use of simpler stimuli within a psychophysical tradition, has demonstrated that perceptual learning can be specific to particular stimulus features (e.g., size [11]; orientation [12]; texture [13]; retinotopic location [14]; for reviews involving perceptual learning in other modalities, see [15], [16]). In addition to the nature of the stimuli that are presented, it is also notable that experiments from the psychophysical tradition typically involve extended stimulus exposure and/or training over a period of hours or days, with feedback to the participant. Whereas those influenced by the associative tradition, (e.g., [5]–[10], [17]), typically involve less extended exposure, without explicit feedback. Despite these apparent differences, results from both methodological backgrounds reveal the benefit of stimulus exposure on subsequent perception, and have proposed similar mechanisms to explain this learning [1]–[3], [18]. For an extended analysis of these two traditions and the relationships and differences between them see Dwyer and Mundy [18].

While there has been little work on the brain correlates of experience-dependent improvements in discrimination from within an associative tradition, the stimulus specificity effects from within the psychophysical tradition [11]–[16] are consistent with the view that perceptual learning is based upon changes in primary sensory cortex where receptive fields are relatively small and feature specific (but see, [19]–[21]). This view receives direct support from functional imaging studies that have implicated early visual regions in perceptual learning. For example, Schiltz and colleagues [22], using PET, reported a reduction in activation in visual cortex following extended training with contrast discrimination. Similarly, Mukai and colleagues [23], in an fMRI study, found a decrease in activity in the visual cortex after training with sinusoidal gratings (there were also changes in the activity of “attentional” regions such as frontal and supplementary eye-fields and dorsolateral pre-frontal cortex). In this latter study, participants who improved on the discrimination task (‘learners’) showed a reduction in visual cortex activation (and areas belonging to the attentional network) that correlated with the magnitude of perceptual learning. In contrast, participants who failed to improve on the task (‘nonlearners’) showed no changes in brain activation during learning.

The studies described above highlight a role for visual cortex in perceptual learning that is evident as reductions in activity after training (see also [24]). However, other studies have observed increases in activity for exposed or trained stimuli versus novel stimuli (1 month training, low contrast patterns, early visual cortex increase [25]; 24 hours training, texture discrimination, early visual cortex increase [26]). The basis for these opposing changes in activation in the primary visual cortex after stimulus exposure is unclear. However, one potential explanation relies on the assumption that these studies have measured different regions in the visual cortex, which might differentially change as a consequence of experience. We investigated this explanation, in terms of anatomical specificity, by assessing different regions of visual cortex with retinotopic mapping, and examining whether or not any changes in activity therein relate to the degree of discrimination improvement produced by stimulus exposure (see [23]). Moreover, as noted above associative and psychophysical investigations of perceptual learning typically differ with respect to the overall amount of exposure and the complexity of the stimuli. By using fMRI with relatively brief exposure to complex stimuli we will be able to assess whether the neural sequelae of this type of experience are related to discrimination improvement in the same way as with extended exposure to simple stimuli.

A complimentary aim of this study was to examine the extent to which any changes (increases or decreases) in activation were related to stimulus familiarity *per se*. One simple explanation for perceptual learning, which stems from within an associative tradition, relies on the idea that it is a direct function of the frequency with which the stimuli are encountered (i.e., their familiarity [3], [28]). However, this idea cannot explain the fact that different *schedules* of exposure (that match for the overall *amount* of exposure) result in differences in the improvement in discrimination. For example, Honey, Bateson and Horn ([29]; see also [6]) demonstrated that animals exposed to two stimuli in an intermixed fashion (i.e. A, A*, A, A*, …) subsequently acquired a discrimination between them more readily than a second group of animals that received an equivalent amount of exposure to the stimuli but arranged in blocks (i.e. A, A, … A*, A*,). Such effects of exposure schedule have since been replicated in animal and human studies across a range of stimuli, demonstrating that the experience-dependent changes in discrimination cannot be solely a product of stimuli familiarity (for a recent review, see [30]). The theoretical basis of such a scheduling effect remains a matter of debate (see below); and this debate is mirrored by the fact that we do not know whether the improvements in discrimination that are produced by different types of stimulus exposure are mediated by the same brain substrates.

One of the earliest theoretical accounts of perceptual learning, that anticipated the advantage of intermixed over blocked exposure, was provided by Gibson [4], [27]. She suggested that perceptual learning depends on the process of stimulus differentiation; more specifically, the effectiveness of the features that are unique to each of the exposed stimuli are enhanced relative to those features that are shared or common to both. More recently, we have suggested that this differentiation process might be understood in terms of the differential adaptation of stimulus-unique and stimulus-common features (e.g. [7], [31], [32]; but see also [33], [34]). Briefly, according to this analysis, the response to common elements will adapt more quickly than the response to unique features because the common features are present on every trial. This difference will result in the unique features being better attended to and processed and will, in turn, result in them being more readily integrated into a long-term representation of the stimulus. Although the evidence suggesting an interaction between adaptation and exposure-dependant improvements in discrimination in our studies is relatively indirect, there is now more direct evidence to support such an interaction from motion-direction discrimination tasks [35]. The fact that the schedule of exposure has an equivalent effect on the improvement in discrimination across a range of stimuli might be taken to imply that shared brain mechanisms are involved (see [9]). Although some preliminary evidence suggests that common brain mechanisms are involved across different types of visual stimuli [17], this study examined only two stimulus types (faces and checkerboards) and involved too few participants to afford a powerful analysis of brain-behaviour relationships. Thus, the final aim for the current study was to examine the extent to which improvements in discrimination involving different classes of visual stimulus is supported by shared brain mechanisms.

To summarise: The three main aims of this study were to: (i) use retinotopic mapping techniques to determine the involvement of sub-regions within the visual cortex in the discrimination improvement following brief exposure to complex stimuli; (ii) investigate whether the schedule of exposure affects the brain mechanisms recruited; and (iii) examine the extent to which the brain mechanisms (in visual cortex and elsewhere) recruited are common across different types of visual stimuli. The design of the study is summarized in Table 1. It involved giving participants intermixed exposure to two pairs of similar stimuli and blocked exposure to a further two pairs. The effects of this unsupervised exposure training were tested by examining the ability to discriminate within the intermixed and blocked pairs, in comparison to the ability to discriminate within two novel pairs of stimuli. This procedure was repeated with dot stimuli, morphed faces and virtual reality scenes. MRI data acquisition was performed during the test phase with a retinotopic mapping sequence performed in a separate session. Contrasting intermixed exposure with novel stimuli provides an assessment of experience-dependent improvement in discrimination. In this case, the effect of experience is based upon a within-subjects comparison, but is evident as a difference in performance between stimuli (exposed and novel). Contrasting the improvement in discrimination between stimuli experienced in intermixed and blocked schedules assesses effects of experience over and above those that reflect mere familiarity. Comparison of the three types of stimuli (for both the intermixed vs novel, and intermixed vs blocked comparisons) assesses the degree to which the brain mechanisms recruited by experience are stimulus general. Finally, correlating the behavioural effects of stimulus exposure on test performance with the fMRI results allows assessment of the relationship between the degree of experience-dependent improvement in discrimination and key brain substrates.

**Table 1:** Experimental design.

Condition	Exposure	Discrimination
Intermixed	A, A*, A, A*, A, A*, A, A*, A, A*,	A versus A*
	B, B*, B, B*, B, B*, B, B*, B, B*	B versus B*
Blocked	C, C, C, C, C, C*, C*, C*, C*, C*,	C versus C*
	D, D, D, D, D, D*, D*, D*, D*, D*	D versus D*
Control	No Exposure	E versus E*/F versus F*

Note: A/A* to E/E* represent pairs of difficult to discriminate stimuli. A within-subjects factorial design was used that manipulated exposure type (intermixed, blocked, and control) and stimulus type (dots, faces, scenes). Each presented image was shown 5 times for 2 seconds each with a 1 second ISI. After an exposure stage (A/A* and B/B* intermixed, C/C* and D/D* blocked), participants received a same/different test phase in which the exposed stimuli and two novel pairs of stimuli (E/E* and F/F*) were presented. This design was repeated six times (twice each with dots, faces, or scenes) with different stimuli as A–F.

## Materials and Methods

### Participants

Sixteen right-handed healthy participants (10 male) were scanned. Participants ranged from 18 to 40 years old (mean = 30.1 years) and all had normal or corrected-to-normal vision. This work received ethical approval from the School of Psychology (Cardiff University) ethics committee. All participants gave informed, written consent and were paid for their participation.

### Materials

**Figure 1:**
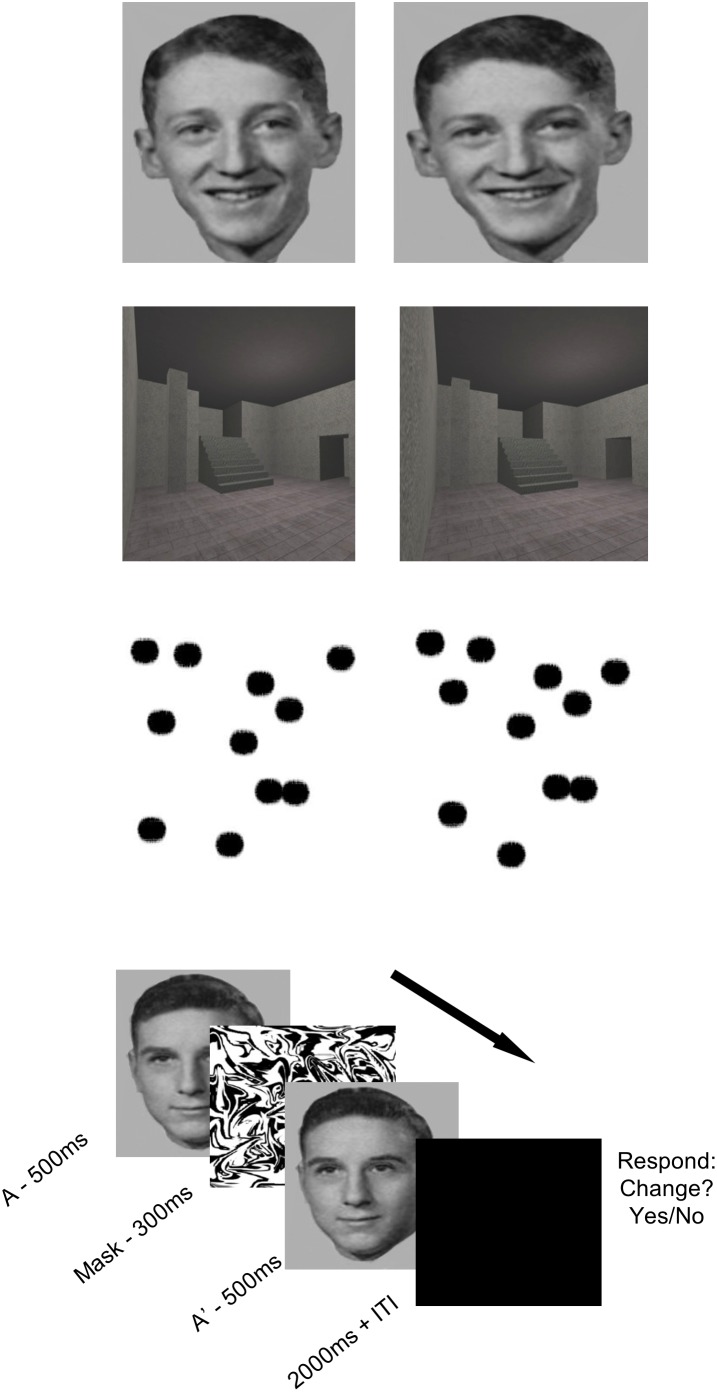
An example of the face-, dot-, and scene-pair stimuli used in the main experiment. The on-screen dimension of all images was 15×12 degrees of visual angle (height × width), with a fixation distance of 57 cm. The lower panel depicts the sequence of a single test trial.

Dot patterns, faces and scenes were used in both the pre-exposure and discrimination conditions. A computer program, written in Visual Basic^©^, was used to generate twelve pairs of confusable dot patterns. The program was constrained to create an initial random pattern of 11 dots. A second confusable pattern was made by making random adjustments to the location of 4 dots in the original image within a range of 50 pixels. The face stimuli were created using a morphing software package called Morpheus 1.85 (ACD Systems, Saanichiton, British Columbia, Canada) running on an IBM-compatible PC. Twelve face pairs (6 pairs of men and 6 pairs of women) were created by selecting exemplars that were close together on a morph continuum between photos of two different individuals. This process is reported in more detail in Mundy et al. [7]. The scene stimuli consisted of twelve pairs of computer-generated virtual rooms. The pairs of rooms were made confusable by creating each pair from the same prototype, but ensuring that within the pair there were differences in the size, orientation and/or location of one or more of the features of the room (e.g. a window, staircase, wall cavity). The rooms were created using a commercially available computer game (Deus Ex, Ion Storm L.P., Austin, TX, USA) and a freeware software editor (Deus Ex Software Development Kit v1112f). Further details on how both faces and scenes were presented on screen are identical to those reported in Mundy et al. [10]. An example of the stimuli used can be found in Figure 1. All stimuli were presented at the centre of the screen; the on-screen dimension of all images was 15×12 degrees of visual angle (height × width), with a fixation distance of 57 cm. Participants were asked to fixate centrally, aided by a crosshair presented during inter-trial intervals. Response times and accuracy were automatically recorded via button box in order that individual trials could be accurately classified for an event-related analysis of the fMRI data.

### Experimental Design & Procedure

The experimental design is summarized in Table 1. This design was run six times, twice with each type of stimulus (dots, faces and scenes), with stimulus-type order counterbalanced across participants. This arrangement was split across two scanning acquisition runs, each containing one version of each stimulus type. The twelve stimulus pairs produced for each condition were therefore split into two batches of six stimulus pairs. The stimulus batch was changed between experiment repetitions and counterbalanced across participants so that all stimulus pairs appeared equally often in each condition (intermixed, blocked and novel). For each stimulus type, the experiment took place in two stages: ‘exposure’, where participants were given experience of some stimulus pairs; and ‘test’, where discrimination between the members of exposed and novel-at-test pairs was assessed. Before exposure, participants were given the following instructions: “You will now see a series of images, some will be very similar, please play close attention–the differences are very subtle. Please try and keep your gaze to the centre of the screen (indicated by a crosshair)”. During the exposure stage (given in the scanner but with no data acquired), participants were given exposure to pairs of stimuli in two exposure-schedule conditions. In the intermixed condition, the two images in a pair were presented in alternation. In the blocked condition, two pairs of items were presented, with all presentations of one image in the pair preceded by those of the second image in that pair. The order in which these within-subjects conditions were presented was counterbalanced across participants and runs. Each presented image was shown 5 times for 2 seconds each with a 1 second ISI. This amount of exposure produces differences in discrimination between exposed and novel stimuli and between stimuli presented on intermixed and clocked schedules (e.g. [7], [9], [17], [31]). The remaining two pairs of items in each stimulus condition were only presented during the test stage of the experiment, and were thus ‘novel’ at the outset of testing.

The test phase, which immediately followed exposure, consisted of a same/different discrimination task in which two stimuli were presented in succession: on each trial they were either two copies of the same stimulus (i.e. A, A) or two different stimuli (from the same pair, i.e. A*, A) and the participants were requested to indicate if they were the same or not by pressing the relevant key of the response box. Participants saw the following instructions before the test phase commenced: “You will now see a second series of faces, some will be new. The image will flash–please indicate whether you think the image has changed. Press the left button to indicate that you saw the images change (i.e., they were different). Press the right button to indicate that the images did not change (i.e., they were the same)”. The first stimulus was shown for 500 ms, followed by a 300 ms ISI that was filled by a high contrast random mask image, and then the second stimulus was presented for 500 ms. The offset of the second stimulus triggered a 2-sec response window (when the screen was blank), followed by a random ITI of between 2.5 and 10 sec sampled from a Poisson distribution, during which time a central fixation cross was presented. The test phase was conducted with 16 trials for each stimulus-pair (8 same, 8 different). The order of trials was randomised with the constraint that there must be four trials from each condition in every 16 trials. After every 16 trials, a fixation cross appeared for 20 seconds to allow the participant to rest. Following the main experiment, each participant was given a high-resolution structural scan. Additionally, 12 of the 16 participants underwent a retinotopic mapping sequence (for details of this protocol, see [37], [38]), undertaken in a second scanning session between 1 and 7 days later. The 4 participants who did not receive a retinotopic scan were unavailable to return for this second session. These participants were thus excluded from retinotopic analyses, but were included in all other analyses.

### Data Acquisition

Imaging was performed on a General Electric 3-T HDx MRI system using an 8-channel receive-only head coil. For functional imaging a T2*-weighted gradient-echo, echo-planar imaging (EPI) sequence was used to image volumes with blood oxygen level dependent (BOLD) contrast. Fifty slices were collected per image volume covering the whole brain, prescribed 30 degrees inclined from the AC-PC plane. Scanning parameters were: repetition time/echo time (TR/TE) 3,000/35 ms; flip angle (FA) 90 degrees; slice thickness 2.8 mm (1 mm gap); acquisition matrix GE-EPI 64×64; in-plane field of view 22 cm; ASSET (acceleration factor) 2. Additional high-resolution field maps were also acquired for every participant, for the purpose of un-distorting the EPI datasets during image pre-processing. For anatomic localization, a structural scan was made for each participant using a T1-weighted sequence (3D FSPGR). Scanning parameters were: TR/TE 7.9/3.0 ms; FA 20 degrees; acquisition matrix 256×256×176, field of view 256×256×176 mm, 1 mm isotropic resolution.

### Data Pre-processing

Data pre-processing and statistical analysis of fMRI data was carried out using FEAT (FMRI Expert Analysis Tool) Version 5.63, part of FSL (FMRIB’s Software Library, www.fmrib.ox.ac.uk/fsl). The following pre-statistics processing was applied; motion correction using MCFLIRT [39]; non-brain removal using BET [40]; spatial smoothing using a Gaussian kernel of FWHM 4 mm; mean-based intensity normalisation of all volumes by the same factor; highpass temporal filtering (Gaussian-weighted least-squares straight line fitting, with sigma = 20.0 s); un-distorting the EPI data to correct for magnetic field distortions by means of individual fieldmaps. Time-series statistical analysis was carried out using FILM with local autocorrelation correction [41]. Registration to high resolution 3D anatomical T1 scans (per participant) and to a standard Montreal Neurological Institute (MNI) template image (for group average) were carried out using FLIRT [39], [42].

### Data Analysis

#### Behavioural Analysis

The primary measure of performance was response accuracy (percentage of correct responses during discrimination testing). This was calculated on a within-subject basis as a function of exposure type (intermixed, blocked, novel) and stimulus type (faces, dots, scenes) averaged over both scanning runs. In order to assess any effects of the speed of responding (e.g., speed accuracy trade-offs), reaction times were also examined in the same manner.

#### Imaging: General Group Analysis

After pre-processing each individual subject’s fMRI time series, the data were submitted to a (random effects) general linear model, with one predictor that was convolved with a standard model of the haemodynamic response function (HRF) for each event-type/condition. Only data from the discrimination test phase were analysed: consequently, the event-types/regressors (a total of 9) were defined by the exposure history of each discrimination event (‘intermixed dots’; ‘blocked dots’; ‘novel dots’; ‘intermixed faces’; ‘blocked faces’; ‘novel faces’; ‘intermixed scenes’; ‘blocked scenes’ and ‘novel scenes’). The parameter estimates relating to the height of the HRF response to each event-type were calculated on a voxel by voxel basis, via a multiple linear regression of the response time course, to create one beta image for each event-type per participant, per run. These parameter estimates, characterising the extent to which a region was activated by the event-type, were used as the basis for our analyses by including them in a higher-level (group) FLAME analysis (FMRIB’s Local Analysis of Mixed Effects [43], [44]).

#### Planned data analyses

First, BOLD activity resulting from stimulus independent exposure learning was defined in a whole-brain analysis by contrasting intermixed stimuli (intermixed dots + intermixed faces + intermixed scenes) with novel-at-test stimuli (novel dots + novel faces + novel scenes), for each individual. Then, FEAT’s (gaussianised) *t*-statistics were converted to *z* statistics and thresholded using clusters determined by *z*>3 and a (corrected) cluster significance threshold of *p* = 0.05 [45]. To test for any stimulus-specific effects, the same whole-brain analysis was then repeated for each of the stimulus types individually. To obtain a group average for each of these contrasts, the data were submitted to a further FLAME analysis.

To assess the contribution of distinct early visual regions to the effects of stimulus exposure, and to allow a more detailed assessment of which early visual areas may contribute to degree of learning, the individual retinotopic maps (N = 12) were scrutinised to identify areas V1–V4 in each hemisphere (see [37], [38]). Since all stimuli were centrally presented, the centre of each region (close to the foveal representation) was identified by eye, according to careful analysis of the pattern of striation in each individual’s retinotopic map. The map was represented as a cortical flatmap with areas of activity delineated by selectivity to visual field meridian. The voxel closest to this point was used to define the centre point for a subsequent manually defined region of interest (ROI) constructed from the set of contiguous voxels within 6 mm in the anterior/posterior, superior/inferior and medial/lateral direction of this co-ordinate. Where retinotopic regions were further sub-divided by the retinotopic map (i.e. V2dorsal/V2ventral, V3/V3a/V3b/Vp), ROIs were defined for each sub-division and data were later averaged across them. Voxel numbers in each ROI were matched. Using these retinotopic ROIs, each participant’s individual data was queried to obtain a parameter estimate in each of the visual regions within the exposure contrast [intermixed stimuli versus novel stimuli] for each stimulus type, in each hemisphere.

In order to examine exposure effects in previously reported frontoparietal areas (e.g., [17], [23], [46]), and to evaluate those regions identified in our own whole-brain analyses, several candidate regions were defined anatomically in reference to the atlas of Talairach and Tournoux [47] in all 16 participants: intraparietal sulcus (IPS; Talairach coordinates x, y, z: ±26, −65, 39), frontal eye field (FEF; ±41, 2, 47), supplementary eye field (SEF; ±3, −1, 60), and dorsolateral prefrontal cortex (DLPFC; ±23, 42, 36). The subsequent ROI for each region was defined as the set of contiguous voxels that were within 6 mm in the anterior/posterior, superior/inferior and medial/lateral directions of voxel closest to the anatomical centre (i.e., a 3×3×3 voxel cube). The validity of such regions to areas of activity seen in our own whole-brain data was assured by cross-checking the proximity of the central voxel of these anatomically defined ROIs with the peak voxel of clusters identified within the above whole-brain analysis, to ensure good correspondence. A series of *t*-tests supported this correspondence, by indicating that anatomically defined regions did not differ significantly in any (x, y, z) direction (maximum *t* (15) = 1.75, *p*>0.1) from significant clusters seen in group-average whole brain analyses. Using these anatomical ROIs, as before, each participant’s individual data was queried to obtain a parameter estimate in each of these regions within the learning contrast [intermixed stimuli versus novel stimuli] for each stimulus type, in each hemisphere.

In order to investigate the possibility that activity in brain regions resulting from exposure to the stimuli might be modulated by individual differences in discrimination improvement, correlations between individual parameter estimates and a behavioural measure of discrimination improvement were made for each frontoparietal and visual ROI. The behavioural measure was defined as an individual’s score on exposed intermixed discriminations minus their score on novel discriminations; thus a larger difference equates to greater improvement in stimulus discrimination (a greater benefit to performance of exposure compared to a no exposure baseline). For the subsequent analysis of the effect of exposure schedule on discrimination improvement (as opposed to the amount of exposure), the above methodology was repeated in full, replacing novel stimuli with blocked stimuli. In this way, all contrasts became a test of the sequence of presentation [intermixed stimuli versus blocked stimuli] rather than the amount of presentation (i.e., exposed versus novel). It should be noted that all these analyses make comparisons across different exposure conditions. We have already reported the results of an, entirely orthogonal, analysis based on differences within exposure conditions and stimulus-types [10].

## Results

### Behavioural Data

**Figure 2:**
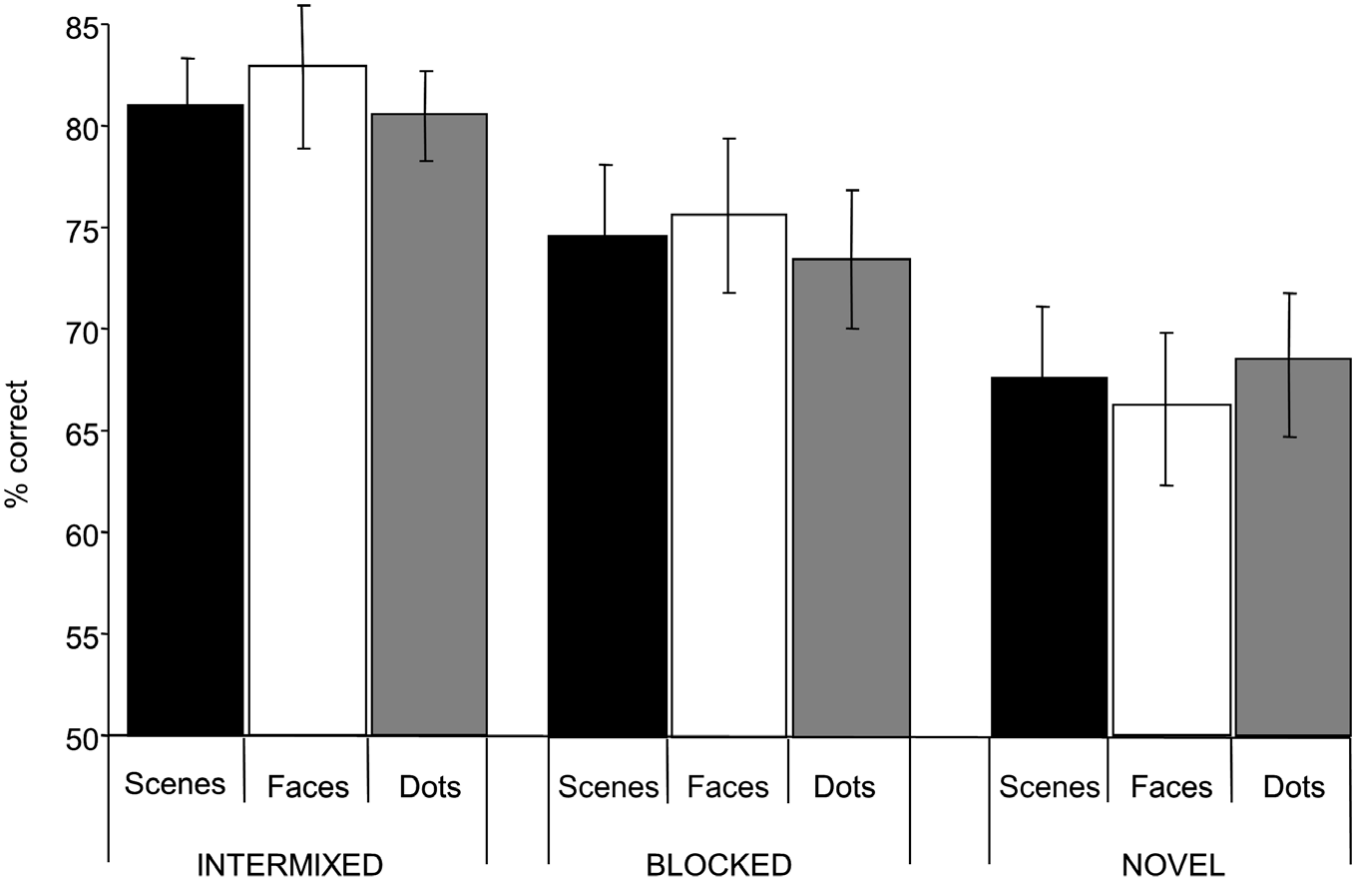
Mean discrimination performance (with SEM) as percentages correct. Scenes (black), face (stripe) and dots (white) refer to the nature of the stimulus, whilst Intermixed, Blocked and Novel refer to the exposure status.

Figure 2 shows the discrimination scores for the nine conditions. Discriminations involving pairs of images that were exposed in an intermixed (and blocked) fashion were more accurate than those involving pairs of images that were novel. Similarly, intermixed exposure resulted in better performance than blocked. There was, however, little evidence of performance differences on discriminations across different stimuli type. Consistent with this description of the results, analysis of variance revealed a main effect of exposure condition (*F*(2, 30) = 12.9, *p<*0.01), but no overall effect of stimulus category (*F*<1) and no interaction (*F*<1). Collapsing across stimuli, analysis of the simple main effects confirmed that performance in each of the two exposed conditions was better than in the novel condition (minimum *F*(1,15) = 31.2, *p*<0.01), and that performance in the intermixed condition was more proficient than in the blocked condition (*F*(1,15) = 14.9, *p*<0.01). Additionally, when the behavioural data were split, so that performance in the first half of the test phase was compared with performance in the second half (i.e., a broad examination of learning across test) ANOVA revealed a main effect of test phase (early vs late *F*(1, 15) = 22.7, *p*<0.01), but this did not interact with exposure schedule (*F*<1). Investigating a comparable split half analysis in our imaging data was not possible due to lack of power for this kind of contrast, however given the behavioral data indicate no interaction between the main effects of test phase and schedule it would appear that the effects of exposure gained at test are simply added to the effects of the explicit manipulation of exposure in the previous stage of the experiments.

Mean reaction time data for this experiment can be found in Table 2. ANOVA revealed no significant effect of exposure or stimuli type and no interaction (*Fs*<1). These behavioural results parallel those seen by Mundy et al., [5], [7], [9], [17] and elsewhere (e.g., [6], [8], [29], [36]).

**Table 2:** Behavioural reaction time data for each condition (seconds), with SEM.

	Intermixed	Blocked	Novel
**Scene**	1.97 (0.221)	1.95 (0.206)	1.90 (0.234)
**Face**	1.85 (0.194)	1.87 (0.222)	1.82 (0.225)
**Dot**	1.89 (0.213)	1.84 (0.199)	1.82 (0.205)

The behavioural data indicating the most marked improvement in discrimination involved the comparison of intermixed and novel stimuli. In order to gain the clearest picture of the brain effects of this experience-dependant improvement in discrimination we began by comparing these two conditions. Later, we turn to the comparison of the intermixed and blocked conditions. Whilst these analyses were the most appropriate for the questions at hand, there were several other potential comparisons: for example, intermixed and blocked conditions can be combined to create an ‘exposed’ condition, which can be contrasted with novel stimuli. This contrast will not be reported in detail, but it is worth noting that there were no significant differences in cortical regions activated, between this comparison and the intermixed versus novel comparison which we report in detail here. As will be described in detail below, with one exception, the general pattern of results from the intermixed versus novel comparison, and intermixed versus blocked comparison, was the same.

### Imaging Data

#### Stimulus independent improvements in discrimination whole brain analysis: Intermixed stimuli versus novel stimuli

**Figure 3:**
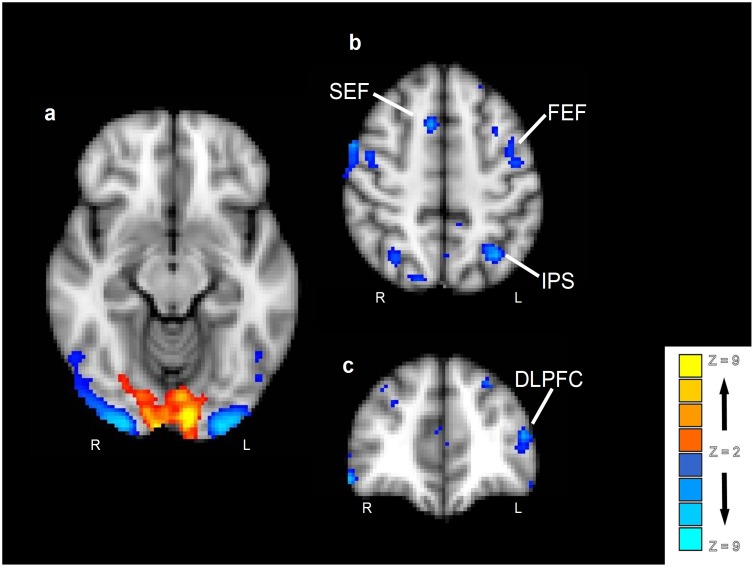
Main effect of intermixed versus novel stimuli. Contrasts in a group analysis (n = 16) were overlaid on the MNI-152 structural standard image. Effects were colour-coded such that intermixed > novel are in red-yellow and novel > intermixed are in blue-lightblue.

In order to examine stimulus-independent improvements in discrimination, data from discriminations involving intermixed stimuli were contrasted with data from discriminations involving the novel stimuli, collapsed across stimulus type (see Figure 3 and Table 3). This contrast revealed a significant area of activation in the occipital pole that extended into the medial inferior occipital gyrus and lingual gyrus (overlapping V1 and V2, according to the Jülich histological atlas in FSL [48]). The reverse contrast revealed significant clusters of activation in: lateral occipital and lingual gyri (overlapping V3 and V4); intraparietal sulcus; superior frontal gyrus (at the junction of the pre-central sulcus, encompassing the frontal eye field); mid frontal gyrus, extending to dorsolateral prefrontal cortex; precuneus; and cingulate gyrus (extending to the upper part of the paracentral sulcus, containing the supplementary eye field). Subsequently, a conjunction analysis was performed in order to confirm that activity in these regions is truly stimulus-independent. Activity to each stimulus type was independently contrasted with baseline activity, resulting in three statistical masks (using a *p*<0.05 threshold within each analysis). Then, a conjunction mask was created by intersecting these three masks, identifying regions that were significantly activated by all three stimulus types (*p*<0.05, corrected for multiple comparsions). This conjunction analysis confirmed the above observations, indicating no differences in the areas reported above when directly comparing experience-dependant discrimination improvement across stimulus types (i.e., examining stimulus-selective processing), indicating these regions are stimulus-independent. However, it must also be noted that other brain regions, further along the ventral visual stream (known to be involved in exposure learning effects in a stimulus-specific way) differed when comparing stimulus types in the current data. For example, learning with face stimuli activated the fusiform gyrus and perirhinal cortex and scene stimuli activated the parahippocampal gyrus and posterior hippocampus. As we have discussed such activations elsewhere (see [10], [17]), we will focus on purely stimulus-independent activity here.

**Table 3:** Clusters surviving a whole-brain correction at *P*<0.05.

			Stereotaxic Coordinates			
Contrast	Region	Z	X (mm)	Y (mm)	Z (mm)	Laterality
INT > NOV	Occipital pole/Lingual gyrus	6.4****	−8	94	8	Left
	Occipital pole/Lingual gyrus	3.8**	14	−96	0	Right
	Temporal pole	3.6**	38	12	−28	Right
	Parahippocampal gyrus	3.2*	32	−4	−26	Right
	Parahippocampal gyrus	3.1*	−28	−4	−26	Left
	Paracingulate gyrus	3.0*	14	16	44	Right
NOV > INT	Occipital fusiform gyrus/Lateral Occipital gyrus	12.1****	−26	−96	−12	Left
	Occipital fusiform gyrus/Lateral Occipital gyrus	11.8****	32	−94	−14	Right
	Precuneus/Posterior cingulate gyrus	6.0****	4	−52	12	Midline
	Superior occipital gyrus	5.5****	34	−82	32	Right
	Posterior fusiform gyrus	5.4***	40	−53	−12	Right
	Anterior hippocampus	5.1***	30	−15	−17	Right
	Posterior fusiform gyrus	5.0***	−40	−55	−10	Left
	Inferior parietal lobule/angular gyrus	4.8***	−42	−58	48	Left
	Parahippocampal gyrus	4.8***	−24	−42	−6	Left
	Parahippocampal gyrus	4.7***	27	−40	−7	Right
	Thalamus, ventral anterior nucleus	4.6***	12	−2	4	Right
	Anterior hippocampus	4.6***	−30	−14	−16	Left
	Temporal occipital fusiform gyrus	4.6***	−46	−60	−18	Left
	Middle temporal gyrus	4.6***	−58	−60	−2	Left
	Perirhinal cortex	4.6***	−26	−8	−24	Left
	Thalamus, medial dorsal nucleus	4.3**	12	−16	4	Right
	Inferior temporal gyrus	4.2**	54	−56	−14	Right
	Superior temporal gyrus/Angular gyrus	4.1**	−46	−58	24	Left
	Perirhinal cortex	4.1**	27	−7	−25	Right
	Superior parietal lobule	4.0**	−22	−44	64	Left
	Temporal occipital fusiform gyrus	4.0**	24	−42	−16	Right
	Posterior hippocampus	4.0**	−22	−28	−8	Left
	Anterior cingulate gyrus	3.9**	4	−2	38	Midline
	Precuneus	3.6**	2	−66	38	Midline
	Cerebellum, inferior semi lunar lobule	3.6**	−30	−78	−44	Left
	Posterior hippocampus	3.6**	26	−32	−10	Right
	Angular gyrus	3.5*	−42	−58	50	Left
	Posterior cingulate gyrus	3.2*	0	−36	24	Midline

****indicates P<0.0001;

***indicates P<0.001;

**indicates P<0.01;

*indicates P<0.05.

INT refers to stimuli that have been preexposed in an intermixed fashion, collapsed across stimuli, NOV refers to stimuli that have not been preexposed. *z* refers to the statistic for each cluster reported within each region.

#### Early visual activations assessed by retinotopic mapping

**Figure 4:**
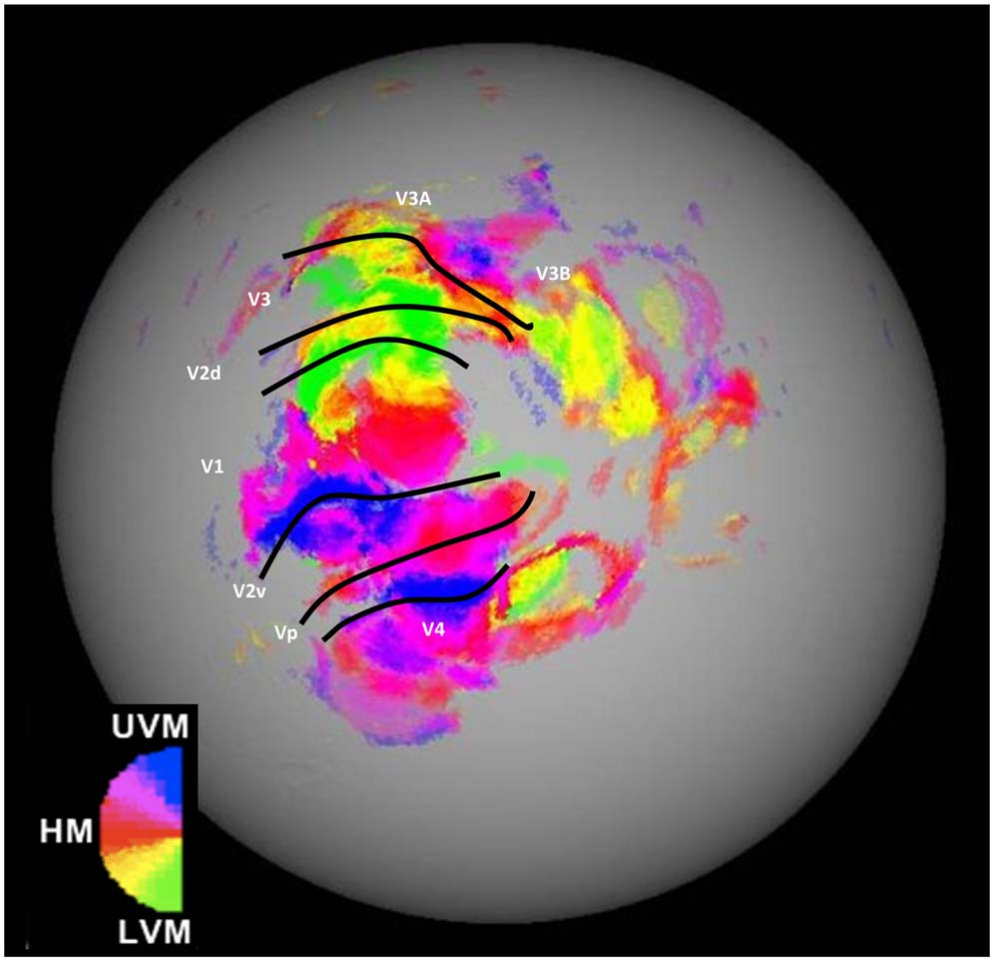
A representative example of a retinotopic map (right hemisphere) from one participant. Retinotopic map colour scheme represents cortical activity to stimuli in the Upper Vertical Meridian (UVM) in blue, through pink, into red coding for the Horizontal Meridian (HM), though yellow, into the Lower Vertical Meridian (LVM) in green.

**Figure 5:**
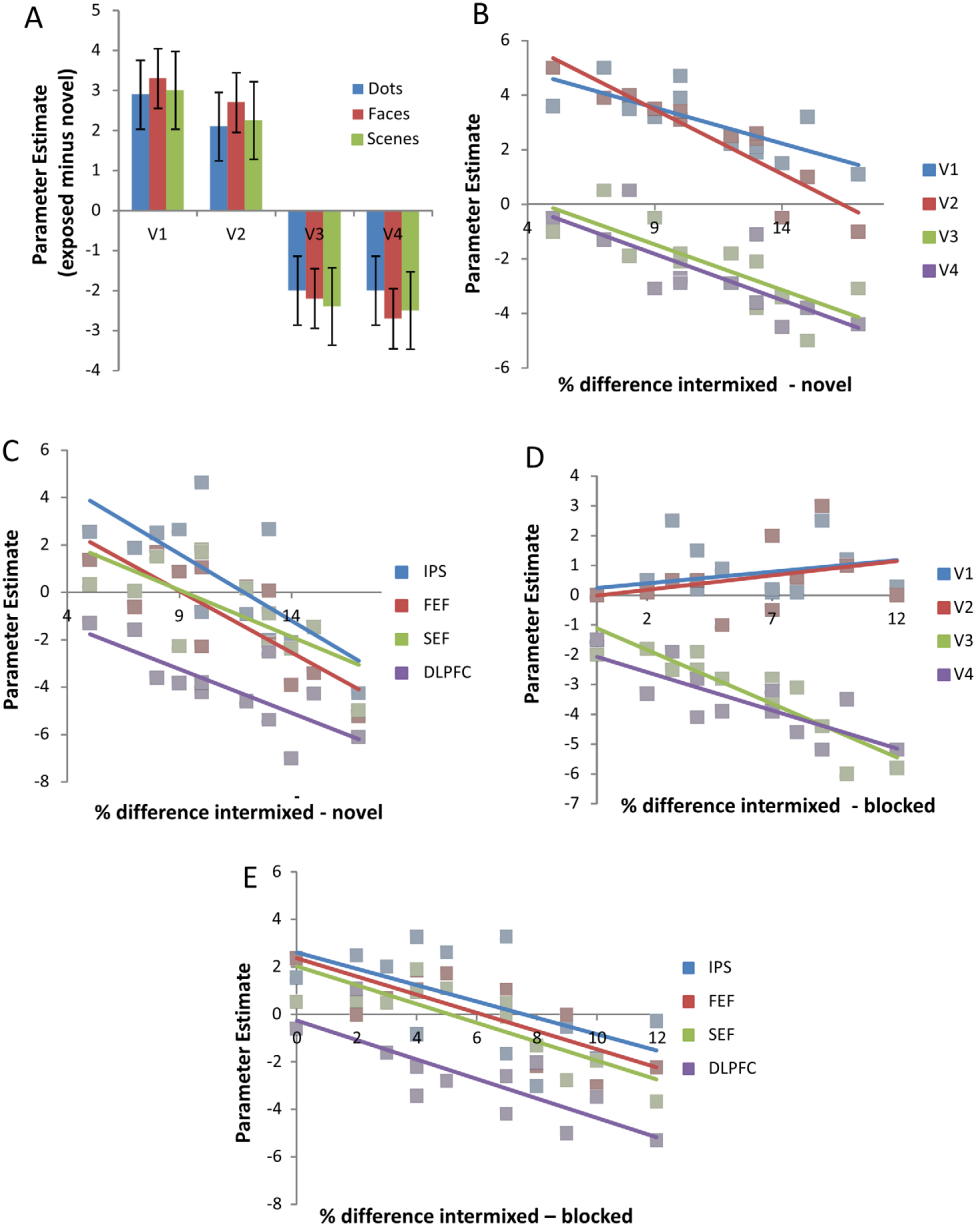
A: The graph shows the activity (as a Parameter Estimate value, error bars represent 95% Confidence Interval) in early visual areas (V1–V4). Activity in V1/2 is significantly above parity between intermixed and novel conditions whereas activity in V3/4 is significantly below. The blue data represents dots, red data faces, green data scenes. B: Correlation between activity and behavioural performance (% difference intermixed discrimination minus novel discrimination) in each of the visual ROIs. C: Correlation between activity and behavioural performance (% difference intermixed discrimination minus novel discrimination) in visual attention related areas. Blue data represents intraparietal sulcus (IPS), red data frontal eye field (FEF), green data supplementary eye field (SEF) and purple data dorsolateral prefrontal cortex (DLPFC). D: Correlation between activity and behavioural performance defined by the difference in performance between exposure schedules (% difference intermixed discrimination minus blocked discrimination) in each of the visual ROIs. E: Correlation between activity and behavioural performance (% difference intermixed discrimination minus blocked discrimination) in visual attention related areas. Blue data represents intraparietal sulcus (IPS), red data frontal eye field (FEF), green data supplementary eye field (SEF) and purple data dorsolateral prefrontal cortex (DLPFC).

Figure 4 shows a retinotopic map for the right hemisphere of a representative participant. The hemisphere is shown with the cortical surface inflated. The most relevant visual sub-regions for this experiment are marked within Figure 4. Regions of interest were defined for each of these sub-regions in both hemispheres of every participant. Figure 5A shows average beta difference scores (i.e., intermixed exposed activity minus activity to novel stimuli) for the exposure contrast in each of the main retinotopic regions, for each stimulus type (collapsed across hemisphere for clarity). Both V1 and V2 respond more to intermixed than novel conditions, whilst V3 and V4 show the opposite pattern. There appears to be no effect of stimulus type on activity in any of the regions. To check that these regions (and thus the relationship between activity and behaviour) were truly stimulus-independent and that there were no hemispheric differences in activity, the beta values [intermixed minus novel] for each early visual ROI were submitted to a three-way ANOVA with factors of hemisphere, subregion and stimulus-type. The ANOVA revealed a significant main effect of sub-region (*F*(3, 33) = 46.2, *p*<0.01), but no effects of stimulus-type or hemisphere or any interactions (*F*s<1).

#### Stimulus independent activity correlated with behavioural performance

In order to gain a better understanding of the activity associated with stimulus independent discrimination improvement, and to assess whether or not it varied according to individual behavioural performance, parameter estimates from the exposure contrast in frontoparietal and early visual regions of interest were correlated with behavioural performance:

*Early visual regions*. Figure 5B shows a correlation between our behavioural measure (X-axis) and activity in each of the visual cortex sub-regions (V1–V4; Y-axis). Inspection of the figure shows that differential exposure-related activity in all regions was negatively correlated with behavioural performance. The greater the difference between intermixed and novel discriminations, the less activity is seen in all measured regions of the visual cortex, albeit from different starting baselines. More specifically, V1 and V2 show greater activity to intermixed-exposed than novel stimuli for participants showing the lowest amount of discrimination improvement, but this activity difference reduces as the level of discrimination improvement increases. Areas V3 and V4 are not differentially activated for participants who show low levels of discrimination improvement, but decreased activity for intermixed-exposed compared with novel stimuli emerged as the level of discrimination improvement increased. Pearson correlations confirm this description of the results: V1, *r* = −0.607, *p*<0.05; V2, *r* = −0.748, *p*<0.01; V3, *r* = −0.754, *p*<0.01; V4, *r* = −0.685, *p*<0.05. To focus on the extremes of the discrimination improvement continuum, if a particular participant was showed only a small improvement in discrimination with exposure, they showed strong activation in V1/2 and little, if any reduction in activity in V3/4 when making discriminations about pre-exposed (versus novel) stimuli. In contrast, participants who displayed large improvements in discrimination with exposure tended to show little V1/2 activation and much greater reduction in activity in V3/4 when making equivalent discriminations.

*Frontoparietal regions.* Regions of interest were defined bilaterally for the intraparietal sulcus (IPS), frontal eye field (FEF), supplementary eye field (SEF) and dorsolateral prefrontal cortex (DLPFC; according to previously published co-ordinates and anatomical landmarks, in the manner described above; see [23]). ANOVA confirmed there were no effects of stimulus-type or hemisphere in each of these regions (*F*s<1), indicating that activity in these areas is stimulus-independent. There was, however, a main effect of exposure [intermixed versus novel] (*F*(3,33) = 43.9 *p*<0.01). This main effect was supported by significant differences between novel and intermixed trials in each of the four ROIs (minimum *F*(1,15) = 15.6 *p*<0.01). Figure 5C shows a correlation between our behavioural measure (accuracy on intermixed trials minus accuracy on novel trials) and activity (contrasting intermixed trials with novel trials) in each of the attention-related ROIs, collapsed across hemisphere. Inspection of the figure shows that all regions are negatively correlated with behavioural performance: The greater the difference between accuracy on exposed and non-exposed discriminations, the greater the reduction in activity is seen in all measured regions. Pearson correlation confirms this analysis: IPS, *r* = −0.684, *p*<0.05; FEF, *r* = −0.825, *p*<0.01; SEF, *r* = −0.692, *p*<0.05; DLPFC, *r* = −0.747, *p*<0.01.

#### The effect of schedule on the neural correlates of discrimination improvement: Intermixed stimuli versus blocked stimuli

The effect of schedule was assessed by contrasting intermixed exposed stimuli with blocked exposed stimuli (i.e., from trials where discrimination is based upon the same amount of exposure to the stimuli, but under differing schedules). This schedule contrast produced no extra areas of activity to the exposure contrast explained above. Both contrasts revealed broadly similar clusters over the whole brain analysis, albeit with lower *z* scores in the intermixed versus blocked compared to intermixed versus novel contrast. A small cluster of voxels in early visual cortex, however, failed to show activation in this schedule contrast, yet were activated in the exposure-based contrast above.

*Early visual regions*: Close inspection of the retinotopic mapping data revealed that this discrepancy appeared to be in areas V1 and V2. An ANOVA on the parameter estimates from each condition in each ROI with factors of contrast (exposure or schedule) and region (V1–4) revealed a main effect of region (*F*(3, 33) = 12.44, *p*<0.01), contrast (*F*(1, 11) = 18.91, *p*<0.01) and an interaction (*F*(3, 33) = 8.35, *p*<0.01). Analysis of the simple effects of this interaction confirmed that in V3 and V4 there was no significant difference between exposure and schedule activity (largest *F = *1.70, *p* = 0.218), whereas V1 and V2 activity differed significantly when comparing exposure and schedule (minimum *F*(2, 10) = 9.73, *p*<0.01).

A correlational analysis between behavioural performance and ROI-generated V1–4 parameter estimates (equivalent to the exposure-based analysis explained above and using identical ROI co-ordinates for each participant) was performed on the schedule data. Behavioural performance was measured, in this case, as the percentage difference between discrimination performance to previously intermixed stimuli and previously blocked stimuli. A large difference can be understood as a strong effect of exposure schedule on subsequent discrimination (i.e., a participant who benefits more from intermixed than blocked exposure, despite the equivalent amount of exposure to the stimuli). Parameter estimates were taken for each early-visual ROI from the image generated by contrasting all intermixed conditions (dots, faces and scenes) with all blocked conditions. Inspection of Figure 5D indicates that whilst V3 and V4 show a negative relationship with behavioural performance (similar to that documented above), V1 and V2 do not; in fact there is instead a weak positive relationship (in contrast to the pattern shown above). Pearson correlations confirm this description of the results: V1, *r* = 0.243, *p* = 0.45; V2, *r* = 0.321, *p* = 0.31; V3, *r* = −0.794, *p*<0.01; V4, *r* = −0.609, *p*<0.05. Thus, for participants who only exhibited a small benefit of intermixed exposure over blocked exposure, at a behavioural level, there was no activation of V1/2 and only weak reductions in activity in V3/4. In contrast, for participants with a larger benefit of intermixed over blocked exposure there were greater reductions in V3/4 activity (i.e., intermixed < blocked), but still little difference in V1/2 activity levels.

*Frontoparietial regions:* Frontoparietal regions of interest, described above, were also directly assessed for schedule effects. ANOVA once again confirmed there were no effects of stimulus-type or hemisphere in each of these regions (largest *F* = 1.40, *p* = 0.291), indicating that schedule-related activity in these areas is stimulus-independent. A further ANOVA on the parameter estimates in each ROI, collapsed across stimulus type and hemisphere, with factors of contrast (exposure or schedule) and region (IPS; FEF; SEF; DLPFC) revealed no significant main effects, or interaction (largest *F* = 1.92, *p* = 0.146). Figure 5E shows a correlation between behaviour (accuracy on intermixed trials minus accuracy on blocked trials) and activity (contrasting intermixed trials with blocked trials) in each of the attention-related ROIs, collapsed across hemisphere. Similarly to the effect of our exposure-based analysis above, inspection of the figure shows that all regions are negatively correlated with behavioural performance. Pearson correlation confirms this analysis: IPS, *r* = −0.606, *p*<0.05; FEF, *r* = −0.601, *p*<0.05; SEF, *r* = −0.670, *p*<0.01; DLPFC, *r* = −0.637, *p*<0.01.

## Discussion

As noted in the introduction, perceptual learning refers to the influence of experience on discrimination and it has been investigated using a wide range of procedures. Both brief and extended exposure to visual stimuli improve later discrimination (i.e., produce a perceptual learning effect). One outstanding issue is whether brief and extended exposure to visual stimuli have the same neuronal sequelae. Here, our principle aim was to identify the contribution made by individual sub-regions within visual cortex (in particular V1-V4) to experience-dependent discrimination improvement, while also investigating a broader network of brain regions that might be important. FMRI was used to scan participants during discrimination judgements involving confusable stimulus-pairs of faces, scenes and dot patterns, which had either been exposed in intermixed or blocked schedules, or were initially novel during test. The experiment we report here uses considerably fewer exposure trials and more complex stimulus types than many previous studies that have reported effects of experience on activity within the visual cortex (e.g., [11]–[16]). We will begin by discussing the implications for the understanding of perceptual learning of the stimulus-independent involvement of the visual cortex in discrimination improvement following the brief amount of exposure. We then consider the involvement of frontal/attentional areas, before moving to consider the effects of schedule, rather than amount, of exposure on discrimination improvement.

### Early visual cortex and stimulus-independent discrimination improvement

Discrimination improvement for all types of exposed stimuli – dot patterns, faces and scenes – was associated with a region of early visual cortex (c.f. [23]). Examining the simple effect of exposure to stimuli, we found important differences in how visual sub-regions were recruited: with increases in activity in V1 and V2 (i.e., greater BOLD signal for stimuli exposed in an intermixed fashion compared with to novel stimuli) but a reduction in activity in V3 and V4 (i.e., greater BOLD signal for novel stimuli than the stimuli exposed intermixed). Activity in these regions showed a negative correlation with behavioural performance, albeit with different starting and finishing points. More specifically, there was more activity in V1 and V2 when participants showed a small degree of exposure-dependant improvement in discrimination (as measured by a small difference between accuracy on trials with intermixed and novel stimuli) and a greater reduction in activation in V3 and V4 when there was a significant benefit of exposure (as measured by a large difference between intermixed and novel trials). As will be discussed later, this pattern of results was, by and large, also apparent when the comparison was between stimuli that has been exposed on the same number of occasions, but according to different schedules (intermixed and blocked).

Experience-related activation in early visual regions with complex stimuli has been reported previously [10], [17], and the current study replicates this finding, demonstrating that this activity in these brain areas is not restricted to simple stimuli (e.g., sinusoidal gratings), extended exposure and/or supervision (e.g., feedback during learning [23]). There have been reports of reductions in activation with learning (e.g. [22]–[24]), as well as other reports of increases in activation with learning (e.g. [25], [26], [49], [50]). The current study confirms that both patterns of results can be seen in the same participants, while performing the same perceptual discriminations. Importantly, the two patterns occur in distinct regions. Although V1 and V2 are more active for group average data for exposed than novel stimuli, there are additional visual regions like V3 and V4 that show reduced activity for exposed stimuli. Closer analysis of this data reveals that while V1–V4 all have the same relationship with changes in discrimination performance–behavioural performance is negatively correlated with activity – the broad differences in activation profile reflect differential starting baselines.

The current data are broadly consistent with Mukai et al. ([23], see also [22], [25], [26]) who also demonstrated a clear relationship between activity in early visual and overall level of performance. Participants for whom there was evidence of learning showed greater activation in these early visual areas, with this pattern decreasing across training. By the end of training, this group showed less early visual cortex activation than the remaining participants who had shown no evidence of learning. The reduced activation in the participants that learned (compared with those who did not learn) is much like the negative correlations between activity and behavioural performance seen in the current experiment. Importantly, however, our data also indicate that visual sub-regions do not all show the same level of initial activity. So, another source of the discrepancies amongst previous studies is the level of behavioural performance: in situations where improvements in discrimination were limited increases in activation might be expected, whereas marked improvements might lead to decreases in activation.

The reason behind the initial difference in activity level between V1/2 and V3/4 is unclear, but the fact that the coding of information changes along the visual pathway provides some basis for speculation. For example, V1 and V2 code for local orientation of contours [51], while V4 codes for local curvature and configuration of orientations, and higher areas (e.g., lateral occipital cortex) processing global configurations and shape [51]. Ahissar and Hochstein [52] propose that perceptual learning (and visual processing more generally) involves a flexible focus on which ever coding system might provide the highest signal to noise ratio for a given task. Combining these ideas leads to the suggestion that the stimuli in this experiment can be discriminated both in terms of very local features (e.g. the positioning of a single dot or orientation of a line in a scene) or by more complex (but still relatively local) features. Using the most simple/local coding system may be inefficient as it might require actively maintaining the representations of a multitude of simple features in order to ascertain if any of them change between images, and thus will have a low signal to noise ratio as most individual features do not actually change. Using the more complex coding system could be more efficient as it requires attending to fewer (but more complex) features, although it may not be the default system to use, particularly early on in learning. As the stimuli become more familiar, however, the visual system may swap from focusing on the coding systems used in V1/2 to those in V3/4, thus resulting in better performance overall. Finally, for this hypothesis to explain the different overall levels of activation across V1–V4 requires the assumption that in V1/2 better performance involves more overall activation (possibly as the result of processing a large number of individual features) whilst in V3/4 it involves less overall activation (possibly by selectively tuning to the configurations relevant to the discrimination). However, whether such a mechanism precedes or results from feedback via stimulus-specific processing known to occur elsewhere in the brain, will be a topic for further experimentation.

### Frontoparietal regions and attention

In addition to visual areas, regions of frontal and parietal cortex associated with attentional processing were also involved in discrimination improvement independently of stimulus type. These areas were the intraparietal sulcus, frontal eye field, supplementary eye field (cortical areas known to be associated with modulating attentional signals; e.g. [53]–[57]) and dorsolateral prefrontal cortex (which plays an important role in the integration of sensory and mnemonic information and working memory; see for a review [58]). These areas are strikingly similar to those found by Mukai et al. [23], a study that used much simpler stimuli. Furthermore, like Mukai et al. the current study found that the degree of learning shown by an individual was negatively correlated with the level of activation seen in these attention-related areas of cortex.

The involvement of attention in perceptual learning (at least with very simple stimuli) has been noted before (e.g., [14], [59]). This involvement is consistent with Gibson’s [4] interpretation of perceptual learning, who proposed that learning is brought about by ‘guided exploratory activity’ through peripheral attention, in order to reveal ‘aspects of potential stimulation’ ([4] pg., 63). These perspectives envisage a top-down influence whereby differences in processing at the level of sensory cortex can be attributed to attentional mechanisms. However, the observed attentional differences could also be a product of bottom-up processes. As discussed in more detail below, differential adaptation or habituation of common and unique elements supported by early sensory regions offers one means to direct attention to the features of the stimuli that are most useful for the discrimination (i.e., attention is drawn to the unique elements following habituation of common elements; e.g., [7], [31], [32]). Irrespective of the theoretical interpretation, the current data suggest that weakly learned stimuli place more demands on attentional regions than do better learned stimuli. Similarly, Mukai et al., [23] shows that initial learning activated attentional regions far more than learning later on in testing. Whilst unable to differentiate between cause and consequence at present, this information reinforces both the general idea that attentional processes are involved in experience-dependent improvements in discrimination, and that attentional demands decrease as discrimination improves.

For reasons of clarity this paper has only focused on those areas traditionally associated with visual perceptual learning of simple stimuli (early visual cortex and frontal cortical regions involved in visual attention e.g., see [23]). It is important to note, however, that in regions beyond those discussed here, stimulus type does indeed affect the location of neural activity, including in the extrastriate cortex and the medial temporal lobe [10], [17]. In this light it should be noted that no further relationships were found between cortical activity outside the early visual cortex and reported visual attention regions discussed here and our measures of discrimination (which make stimulus-independent comparisons between exposure conditions). That said, measures of accuracy *within* an exposure condition did relate to activity in stimulus-dependent effect in MTL regions, a pattern not seen in any other cortical region (including those reported here, see [10]).

### Manipulating exposure schedule

Thus far we have concentrated on examining discrimination improvement as a function of the amount of exposure, by comparing both discrimination performance and brain activity linked to exposed stimuli or to a non-exposed novel baseline. However, as was noted in the introduction, it is also well established that the schedule of exposure is important – in particular, intermixed exposure schedules, which afford the opportunity to compare the to-be-discriminated stimuli, support more improvement in discrimination than do blocked schedules (e.g., [6], [7], [29]). As the amount of stimulus exposure is equivalent in intermixed and blocked exposure, any observed differences in behaviour cannot be attributed to the frequency of exposure to the stimuli (i.e., simple familiarity). However, it remains an open question as to whether the psychological mechanisms responsible for schedule effects differ from those supporting effects based on the amount of exposure. Indeed, the idea that perceptual learning reflects an interaction between differential adaptation to the common and unique features of exposed stimuli and the formation of enduring representations applies equally well to both schedule effects and simple exposure (e.g. [7], [31], [32]).

Consider the fact that during intermixed exposure the interval between presentations of the unique features of two similar stimuli is greater than between those of the common features (which both stimuli share, and thus are seen on each presentation). This difference in the patterning of exposure to the unique and common elements might be a particularly effective means of adapting or habituating the common features of the two stimuli, leaving the unique elements to become better represented and available to be learnt about subsequently. In other words, the operation of short-term adaptation/habituation processes has enduring repercussions for the attentional weighting given to the unique and common features (see also [59]). For blocked, the intervals between features are the same for both unique and common features, so the relative timing cannot contribute to the degree of adaptation, but it remains the case that the features that are common to all stimuli will be encountered more often than features that are unique to one or other stimulus. Thus there are still grounds for the unique features to gain relatively greater weighting in the representation of the stimulus as a whole. Of course, novel stimuli afford neither the opportunity for adaptation to differentially-weight the attention to common and unique features, nor the chance to form an integrated representation of the stimulus as a whole at all. Thus, as well as explaining the effects of exposure schedule, our adaptation-based account also applies to the amount of exposure. In this light it is particularly interesting that the same cortical regions are active when contrasting intermixed stimuli with novel stimuli and when contrasting intermixed and blocked stimuli (excepting V1 and V2). Thus, it seems that the improvement in discrimination based on the amount of exposure to a stimulus is, for the most part, underpinned by the same neural processes as the more specific effect of the schedule of exposure. Of course, the existence of a common brain substrate need not indicate that a single cognitive mechanism underlies perceptual learning, and the lack of V1/2 differential activity following intermixed versus blocked exposure (and the presence of this activity when contrasting intermixed with novel stimuli), points to some level of divergence in brain processing. That said, a common mechanism underpinning effects of schedule and amount of exposure is not incompatible with the current data. Consider our previous speculation that V1/2 are initially involved in local, featural discriminations, but can be superseded once more complex configural information becomes available (perhaps from V3/4 or from upstream feedback via stimulus-specific perceptual processing in extrastriate or medial temporal lobe regions [10], [17]). If these very simple features are present in all stimuli, and they differ only in their amount or location, then only the overall amount of exposure (and not the relative intervals) will produce differential adaptation.

In summary, there is a large degree of commonality between the brain regions recruited as a result of simple exposure, and those recruited by the difference in the schedule of that exposure. Taken alongside the fact that the behavioural effects of manipulating the amount and schedule of exposure are similar (they both produce an improvement in discrimination) suggests the nature of exposure primarily influences the degree rather than the quality or kind of learning. This is not to say that the amount of exposure is the sole determinant of perceptual learning (c.f., [28]) but rather that different schedules of exposure afford the involvement of the cognitive and brain mechanisms supporting perceptual learning to different degrees.

## Summary and Conclusion

The present study investigated the effects of stimulus exposure on the visual cortex and frontoparietal regions, both as a function of amount of exposure (intermixed vs novel), the schedule of exposure (intermixed vs blocked), and also of the type of stimulus (dots, faces, scenes). Experience-dependent changes in activity in early visual cortex was seen for all three types of visual stimuli, and was evident when both intermixed was contrasted with novel stimuli and when intermixed was contrasted with blocked exposure. Areas V1 and V2 were activated in participants showing limited exposure-dependent improvement in discrimination, but there was a decrease in this activity as the level of improvement increased. These relationships were only observed in response to the amount of exposure, but not the schedule of exposure. Relationships involving activity in other brain areas and the amount of improvement based upon exposure showed the same pattern though they were typically stronger for the intermixed/novel contrast than the intermixed/blocked contrast. Areas V3 and V4 were not activated in participants showing weak experience-dependent improvements in discrimination, but became progressively deactivated as the level of such improvement increased. Areas known to be involved in visual attention (e.g., IPS, FEF, SEF) were also shown to have a similar relationship with behavioural performance, supporting the suggestion (see [4], [7]) that changes in attention contribute to perceptual learning. Moreover, the similarity between the neural signature of experience-dependent improvements in discrimination based on the amount and schedule of exposure suggests that the two manipulations have their effects through a shared mechanism.
